# How syllabi relate to outcomes in higher education: A study of syllabi learner-centeredness and grade inequities in STEM

**DOI:** 10.1371/journal.pone.0301331

**Published:** 2024-04-17

**Authors:** Maryam Eslami, Kameryn Denaro, Penelope Collins, Jacklyn M. Sumarsono, Michael Dennin, Brian Sato

**Affiliations:** 1 School of Education, University of California Irvine, Irvine, California, United States of America; 2 Division of Teaching Excellence and Innovation, University of California Irvine, Irvine, California, United States of America; 3 Department of Molecular Biology and Biochemistry, University of California Irvine, Irvine, California, United States of America; 4 Department of Physics, University of California Irvine, Irvine, California, United States of America; UCSI University Kuala Lumpur Campus: UCSI University, ISLAMIC REPUBLIC OF IRAN

## Abstract

Fostering equity in undergraduate science, technology, engineering, and mathematics (STEM) programs can be accomplished by incorporating learner-centered pedagogies, resulting in the closing of opportunity gaps (defined here as the difference in grades earned by minoritized and non-minoritized students). We assessed STEM courses that exhibit small and large opportunity gaps at a minority-serving, research-intensive university, and evaluated the degree to which their syllabi are learner-centered, according to a previously validated rubric. We specifically chose syllabi as they are often the first interaction students have with a course, establish expectations for course policies and practices, and serve as a proxy for the course environment. We found STEM courses with more learner-centered syllabi had smaller opportunity gaps. The syllabus rubric factor that most correlated with smaller gaps was *Power and Control*, which reflects Student’s Role, Outside Resources, and Syllabus Focus. This work highlights the importance of course syllabi as a tool for instructors to create more inclusive classroom environments.

## Introduction

Higher education is one of the most promising paths to social mobility in the United States [1], as university graduates find jobs faster, are more likely to be employed, and have higher salaries [2, 3]. Representation of minoritized populations in higher education is one of the most effective ways to close the wealth gap [1]. In addition to decreasing income inequality, equitable participation and success in post-secondary education increase workplace diversity resulting in a greater range of worldviews, productivity, and innovation [4]. Although the US college enrollment gap, the difference in enrollment rate between minoritized (students who are underrepresented in higher education, including Black, Latinx, Pacific Islander, and indigenous peoples) and non-minoritized (students who are White or Asian) students has shrunk, Black, Latinx, and indigenous students still enter college at lower rates relative to White students [3].

In addition, academic success rates once in college still show stark disparities. In particular, science, technology, engineering, and mathematics (STEM) programs in higher education fail to foster and promote equitable and inclusive learning environments which result in lower retention and graduation rates for women, minoritized, and low-income students [5].

Of primary significance is the well-documented “chilly climate” of STEM fields, which refers to the student perception of faculty being unapproachable and intimidating [6, 7]. In higher education, this chilly climate decreases interactions between students and faculty and increases attrition especially among minoritized students [8, 9]. Research shows minoritized STEM students consistently are awarded lower grades than non-minoritized students, which strongly impacts whether they persist in attaining their intended degrees, switch to another or drop out of college altogether [10].

### Theoretical framework

One way to minimize outcome disparities in STEM is by changing the culture of teaching and learning and redirecting the focus from teaching to learning by embracing a more learner-centered approach when designing curricula [11–14]. A learner-centered pedagogy is rooted in the Constructivist Learning Theory which posits that learning does not happen simply by knowledge-transfer from the instructor to the students; instead, learners actively construct knowledge through experience and social interaction [15]. This research is grounded in three of the fundamental tenets of learner-centeredness and active learning pedagogies. First is that learning occurs in a “community” where students collaborate with their peers and comfortably interact with the instructor [16]. A community is built upon trust that arises from a common understanding of the classroom structure and policies. Second is an environment with shared power between students and instructor, where students have the opportunity to take control of their learning. Sharing “power and control” means empowering students by giving them choice and autonomy while asking them to take responsibility for their learning [17, 18]. In this environment, students are expected to engage in independent investigation and presentation of student-generated knowledge. Last is a learner-centered approach towards “assessment and evaluation” as opposed to a teacher-centered one. Assessment should provide ongoing reciprocal feedback among the students and between the students and the instructor through formative assessment [17, 18]. The purpose of evaluation should be the achievement of learning outcomes (which have to be clearly stated) instead of focusing on grades [17, 18].

One means to increase the learner-centeredness in classrooms is through the implementation of active learning pedagogies [19, 20]. Generally, research shows that active learning strategies in STEM courses are more effective than traditional lectures in increasing student performance and retention [11, 12], particularly for minoritized populations [14]. Active learning practices in STEM courses differentially benefit minoritized students by closing outcome gaps, increasing self-efficacy, and promoting a sense of belonging [13, 14, 21].

### Course syllabi as a measure of learner-centeredness

Instructors can demonstrate the learner-centered nature of their course through the syllabus, which practitioners and researchers view as a representation of the course content and structure [22–25]. Palmer et al. [26 p. 37] define the syllabus as “a physical artifact outlining key structural elements of a course.” Eberly et al. [27] argue that the course syllabus is a contract that discusses classroom pedagogy and norms and has a significant influence on course development. A syllabus can provide a snapshot of the course environment including classroom practices and the teacher’s view towards teaching and learning [22–25, 28]. It has also been demonstrated that syllabi are significant artifacts that drive student decisions in course selection [29]. Further, the tone and language of the syllabus can affect students’ perceptions of the course, potentially impacting their engagement with the course, the instructor, and their peers [30, 31]. This is critical in that low engagement disproportionately affects minoritized students by creating grade inequities, consequently resulting in low persistence in completing their program of study [32].

### Research gap and questions

While researchers have examined the learner-centeredness of course syllabi, there has not been an attempt to link syllabus learner-centeredness to student outcomes, in the same vein as research connecting learner-centered teaching practices and outcome gaps. If learner-centered instructional practices correlate with smaller grade gaps, it is possible that a similar relationship exists between learner-centered syllabi and such gaps.

By examining institutional data, Denaro et al. [33] found that there are pervasive racial opportunity gaps (the difference in course grade awarded to students from minoritized and non-minoritized populations) at the course-instructor level in undergraduate STEM courses. Their work explored the course opportunity gaps (recommended by Teach for America in place of achievement gap as to not blame students for systemic injustices) defined as the difference in the course grade (on a 4.0 scale) received by minoritized and non-minoritized students [33]. They suggested that future research should identify classroom policies or instructor pedagogical practices that might be related to such racial opportunity gaps. As such, the current research expands this work by connecting syllabi characteristics and equity within STEM classrooms. While the syllabus is a representation of classroom practices and policies and not a direct measure of them, it is a document that departments and schools can use to further understand a faculty’s intention to create a learner-centered classroom environment. By analyzing whether specific syllabus characteristics are related to courses with more equitable student outcomes, we can provide recommendations to instructors that have the potential to create more inclusive classroom spaces. Further, this work can inform institutions of what resources should be provided to instructors in regard to their syllabus in terms of faculty training to meaningfully leverage this course tool. Specifically, our research questions are as follows:

1. To what extent is syllabi learner-centeredness related to opportunity gaps in STEM courses?
2. Which specific syllabi characteristics are related to these opportunity gaps and to what extent?

## Methods

This study was approved by the University of California Irvine Institutional Review Board (IRB #3269). Written consent was obtained; however, the data was analyzed anonymously.

### Study population and data collection

Our study population encompassed lower division STEM courses taught between Fall 2015-Winter 2020 at a research-intensive, minority-serving (a US designation for colleges and universities with a significant population of students that come from a minoritized population) west coast university in the United States. The specific course-instructor pairs were determined by first identifying instructors who taught the same lower division STEM course at least three times during the study period. This was done in consideration of the variation in opportunity gap (the difference in the course grade on a 4.0 scale received by minoritized and non-minoritized students) size for each course-instructor pairing. The opportunity gap is denoted as Δ*GP*_*ij*_, where *i* represents the course section (*i* = 1,…,*n*_*i*_) and *j* represents the syllabus for each course-instructor pair. The average grade point difference for the *j*th syllabus is denoted by ${\bar{\Delta GP}}_{j}$.

In total, 218 course-instructor pairs were identified (**Table 1**), with 52 classified as large opportunity gap courses (25th percentile of ${\bar{\Delta GP}}_{j}$ and 58 as small opportunity gap courses (75th percentile of ${\bar{\Delta GP}}_{j}$). We conducted a case-control study with syllabi in the large opportunity gap group designated as cases and syllabi in the small opportunity gap group as controls. The large opportunity gap courses had a mean gap of nearly 0.60 GPA points, which is roughly the difference between a C+ and a C- grade awarded to non-minoritized and minoritized students, respectively. The small opportunity gap courses had a mean gap of only 0.17 GPA points. The distribution of the opportunity gaps can be seen in supplemental **S1 Fig in S1 File**. In our sample, the average opportunity gap was -0.36 with a standard deviation of 0.23 (**Table 2**).

**Table 1:** Course-instructor pair sample by opportunity gap size.

Opportunity Gap	Identified (n)	Responded (n)	Response Rate (%)
Large	52	23	44
Small	58	27	46
Typical (Middle Range)	108	--	--
**Total Eligible for Study**	**110**	**50**	**45**
Number of courses identified	218	--	--

*Note*. Course-instructor pairs that were identified as lower division STEM courses taught within the sample period that were taught by a given instructor at least three times. Identified refers to the resulting sample while responded refers to the instructors who provided syllabi after being contacted by email. The large opportunity gap group was the 25th percentile while the small opportunity gap group represented the 75th percentile.

**Table 2:** Descriptive statistics for the course-instructor pairs that fell in the large and small opportunity gap groups.

	Group		
	Large Opportunity Gap	Small Opportunity Gap	Total
Opportunity Gap			
Size	-0.59 (0.06)	-0.17 (0.12)	-0.36 (0.23)
Instructor Type			
Tenure-track teaching faculty	2 (9%)	3 (11%)	5 (10%)
Non-tenure track teaching faculty	11 (48%)	3 (11%)	14 (28%)
Tenure-track research faculty	10 (43%)	21 (78%)	31 (62%)
Discipline			
Biological Sciences	2 (9%)	2 (7%)	4 (8%)
Engineering	3 (13%)	11 (41%)	14 (28%)
Information and Computer Science	4 (17%)	5 (19%)	9 (18%)
Physical Sciences	14 (61%)	9 (33%)	23 (46%)
Number of students			
Black, Latino/a/x, Pacific Islanders or people indigenous to the US and its territories	59 (35)	42 (26)	50 (32)
White or Asian	121 (71)	93 (61)	106 (67)
Total	180 (105)	135 (85)	156 (96)
Minoritized student representation (%)	33 (5)	32 (7)	32 (6)
Number of times the course was taught by instructor	6 (3)	4 (2)	5 (3)
Total	23	27	50

*Note*. The number and percent are presented for categorical variables and the mean and standard deviation are for quantitative variables.

Course syllabi were requested via email (including two follow-up emails to non-respondents) from the instructors that fell in the case and control populations identified above. This request included the study information sheet. While these instructors were identified as having taught that particular course at least three times, we requested the most recent syllabus from the instructor. Even though we acknowledge that the syllabi may have varied amongst the different iterations of the course taught by a particular instructor, syllabi in general tend to be fairly static tools [34, 35]. We received responses from 50 instructors (a 45% participation rate; 44% participation for the course-instructor pairs with large opportunity gaps and 46% for the course-instructor pairs with small opportunity gaps). The instructors that responded were representative of the population in terms of the distribution of the opportunity gaps. The syllabi were stripped of any identifying names, emails, and course numbers to decrease bias in coding. Descriptive statistics for the two groups are in **Table 2**.

### Measures (rubric for evaluation)

We used a modified version of Cullen and Harris’s [22] rubric to code the collected syllabi. Cullen and Harris’s [22] rubric is a seminal work on syllabus design. The rubric has been used by Richmond et al. [28] and has been tested for reliability and validity. There are 13 items included in the rubric: (1) Accessibility of Teacher, (2) Learning Rationale, (3) Collaboration, (4) Teacher’s Role, (5) Student’s Role, (6) Outside Resources, (7) Syllabus Tone, (8) Syllabus Focus, (9) Grades, (10) Feedback Mechanism, (11) Evaluation, (12) Learning Outcomes, and (13) Revision/Redoing. The detailed rubric item descriptions are provided in supplementary materials (**S1 Table in S1 File).** These rubric items are categorized under three factors **(S1 Table in S1 File)**: (1) *Community*, (2) *Power and Control*, and (3) *Evaluation/Assessment*. According to Cullen and Harris [22], *Community* is the average of rubric items 1–3 and measures the degree to which the syllabus fosters a collaborative environment and creates a sense of relevance for the students to the learning environment. This occurs through the development of trust by offering purposive activities to achieve course objectives. *Power and Control* is the average of rubric items 4–8 and shows the amount of choice and autonomy provided to the students, thereby providing them with responsibility for their learning and a sense of shared power regarding classroom policies and procedures. Finally, *Evaluation/Assessment* is the average of rubric items 9–13 and reflects the integration of ongoing feedback into the course for students’ self-evaluation and instructor’s assessment as to whether the class has achieved the desirable learning outcomes [22].

Syllabus Tone and Syllabus Length (total number of pages) was added to the rubric as well as “teacher-centered” versus “learner-centered” terminologies following Richmond et al. [28]. We acknowledge that the level of teacher-centeredness or learner-centeredness is a spectrum not a binary. That is why our modified rubric is on a 5-point scale (0 to 4) with 0 representing the least learner-centered and 4 representing the most (versus the original 4-point scale, 1–4). We added a category for missing information in a syllabus and assigned a score of zero for that item. Cullen and Harris’s [22] rubric is designed to measure learner-centeredness; therefore, an absence of a learner-centered rubric item indicates the instructor places no value on it in the context of the syllabus.

The supplementary materials include the correlation matrix for the rubric items and factors (**S2 Table in S1 File**). We used the irr package and ltm package in R to compute the interrater reliability and Cronbach’s alpha values [36, 37]. The Cronbach’s alpha is given by:

$$\alpha =\frac{p}{1-p}\left(1-\frac{\sum_{i=1}^{p}\sigma_{y_{i}}^{2}}{\sigma_{x}^{2}}\right),$$

where *p* is the number of items $\sigma_{x}^{2}$ is the variance of the observed total test scores, and $\sigma_{y_{i}}^{2}$

is the variance of the *i*th item. The Bootstrap confidence interval is calculated by taking *B* = 1000 samples with replacement from the data, calculating the Cronbach’s alpha for each Bootstrap sample, and computing the 2.5th and 97.5th quantiles of the estimated Cronbach’s alpha to obtain the 95% Bootstrap confidence interval for the true value of Cronbach’s alpha. The Cronbach’s alpha for the 13 rubric items was acceptable (*α* = 0.750) with a bootstrap 95% confidence interval based on 1000 samples of (0.544, 0.849). For the 3 rubric factors, Cronbach’s alpha was 0.775 with a bootstrap 95% confidence interval based on 1000 samples (0.587, 0.877).

### Syllabus coding

Twenty percent of our study syllabi were reviewed by the entire research team during the initial calibration of the rubric. Training took approximately 3 hours until 100% consensus was achieved. Two members of our research group then evaluated the remaining syllabi (M.E and J.S). The raters scored the syllabi in sets of 10, after which they met to calculate the interrater reliability, go over scoring to reach consensus, and discuss the items in the rubric that brought about high levels of disagreement to refine the rubric guidelines and improve accuracy in scoring. There were only three instances in which the two raters were not able to reach consensus on an item in the rubric. A third rater was asked to evaluate that particular item to resolve the disagreement and reach consensus. Initially, Cohen’s weighted kappa for interrater reliability (IRR) ranged between 0.49 and 0.88 for each rubric item with an average IRR of 0.71. However, after reaching consensus the weighted kappa for interrater reliability increased to between 0.76 and 0.99 for the individual rubric items with an average IRR of 0.90—achieving substantial agreement [38].

### Statistical analyses

We conducted a case-control study to assess the association between syllabus components and the risk of having large opportunity gaps compared to small ones. To address research question 1, we tested if there was a difference between the overall rubric scores and the size of the opportunity gaps; we compared the median rubric score for the two groups using a 2-sample test of medians. To address research question 2, we present evidence of syllabus items, syllabus factors, and the syllabus length that correlate with small opportunity gaps using logistic regression. We modeled the odds of a syllabus being in the small opportunity gap group (compared to a large opportunity gap group). We fit a logistic regression model using the stats [39] and MASS package in R [40]. The assumption of the logistic regression model is that there is a linear relationship between the predictor variables and the log-odds of the event that the syllabus is part of the small opportunity gap group. Assuming we have a sample of *n* independent observations, (*x*_*i*_, *y*_*i*_), we obtain estimates for *β*^*t*^ = (*β*_*t*_,…,*β*_*k*_). Let *Y* be whether or not the syllabus was part of the small opportunity gap group and the probability that the syllabus was part of the small opportunity gap group be *p* = *P*(*Y* = 1) and let *x*^*t*^ = (*x*_1_,…,*x*_*k*_) be the *k* predictors in the model, which is given by:

$$log(\frac{p}{1-p})=\beta_{0}+\beta_{1}x_{1}+\ldots +\beta_{k}x_{k}.$$

In order to understand the relationship between syllabus items, syllabus factors, and syllabus length, we conducted a three-stage analysis. In the first stage, we built a logistic regression model where we include all rubric items and the syllabus length as the covariates. The second stage involved building a model where we include all rubric factors and the syllabus length as the covariates. In the final stage, we performed best subsets logistic regression using the bestglm package in R [41] where we minimized the Akaike Information Criteria (AIC) to choose the best fitting logistic regression model. This procedure is done in two steps. In the first step, we use all possible subsets of covariates (syllabus items and syllabus length) and then we calculate the corresponding AIC for each model. In the second step, we carried out best subsets logistic regression using the syllabus items from the first stage and the syllabus factors to obtain the final model. We compare our final model to the results of a stepwise logistic regression for the syllabus items, syllabus factors, and syllabus length. When interpreting regression coefficients from the logistic regression model, one must first exponentiate the coefficients to obtain an estimate for the odds ratio. If we compare two groups of syllabi, with one group having 1-point higher score (more learner-centered) on a particular syllabus item the estimated odds ratio is the increase in the likelihood of the syllabus being from a course with small opportunity gaps.

## Results

### Research question 1: To what extent is syllabi learner-centeredness related to opportunity gaps in STEM courses?

**Fig 1:**
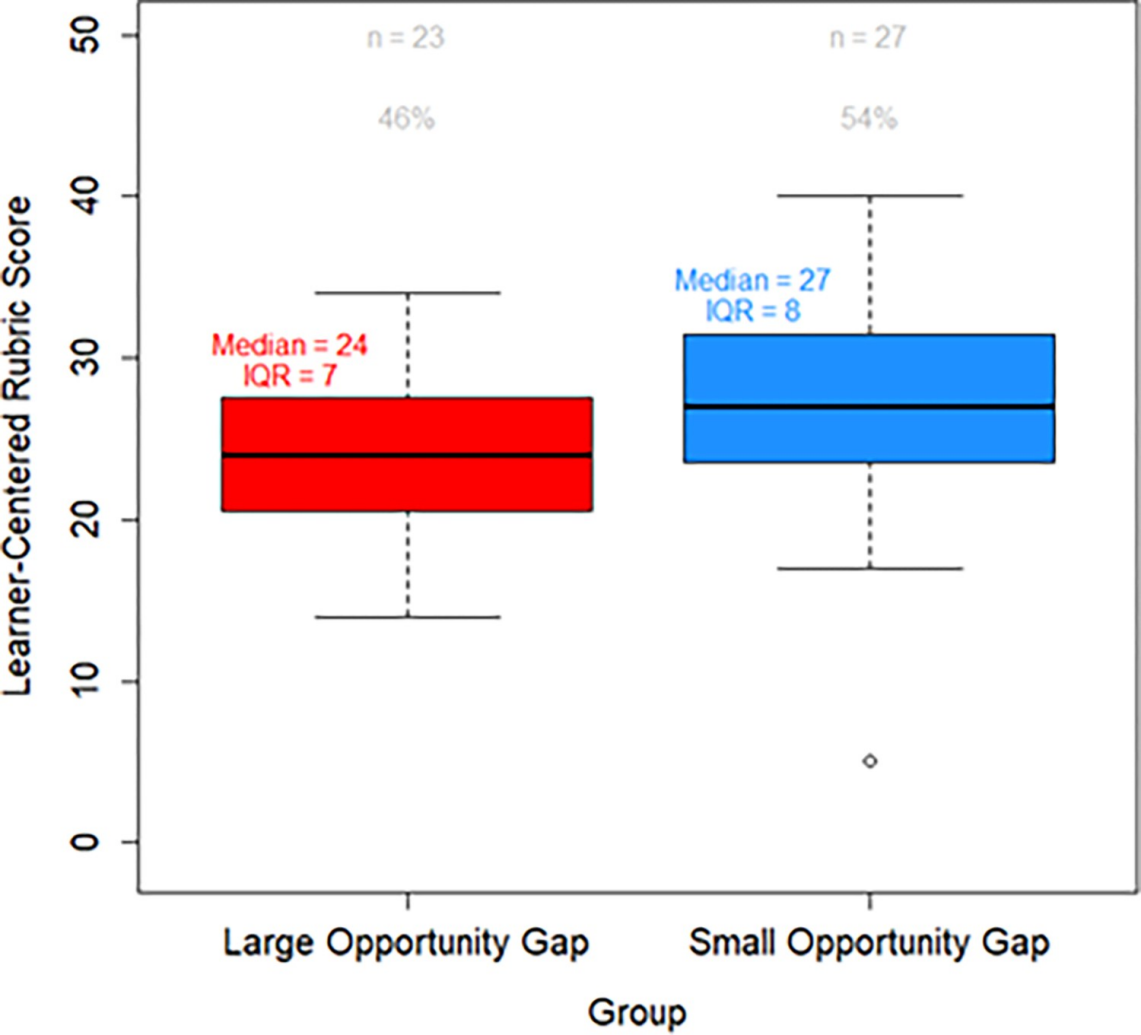
Boxplot comparing learner-centered rubric scores of the small opportunity gap group and the large opportunity gap group. *Note*. The above boxplot displays the distribution of the rubric scores assigned to the course syllabi in both the large (n = 23, 46% of the courses) and small (n = 27, 54% of the courses) opportunity gap groups. Each boxplot contains the minimum, 25th percentile, 50th percentile (median), 75th percentile, and maximum. Outliers are denoted with an open circle and when outliers are present, the whisker reaches to the next value that is not an outlier. The box represents the middle 50% of the data and spans the interquartile range (IQR). The IQR is defined to be the difference between the 75th percentile and the 25th percentile.

We compared the syllabi rubric scores between the small and large opportunity gap course-instructor groups. **Fig 1** shows that the total rubric score is significantly higher (more learner-centered) for the syllabi from the small opportunity gap group (*Δ*_*medians*_ = −3.00, *p* = 0.037). In other words, the course-instructor pairs with small opportunity gaps tend to have more learner-centered syllabi.

### Research question 2: Which specific syllabi characteristics are related to these opportunity gaps and to what extent?

The summary statistics (mean, median, interquartile range (IQR), and standard deviation) of the thirteen individual rubric items, three factors, and syllabus length for syllabi from course-instructor pairs with large and small opportunity gaps are presented in **S3 Table in S1 File**.

**Table 3** is the result of the first stage of our analytic process which predicts the log odds of a syllabus coming from the small opportunity gap group modeled on the 13 syllabus items. When considering all of the individual rubric items at once, none are significant in predicting which syllabi come from the small opportunity gap group.

**Table 3:** Model 1. Logistic regression with syllabus items and syllabus length as covariates.

	Exponentiated Coefficients	95% Confidence Interval for the Odds Ratio	Test Statistic	p-value
(Intercept)	0.31	(-0.52, 0.6)	-0.52	0.6008
Accessibility of Teacher	0.95	(-0.13, 0.89)	-0.13	0.8937
Learning Rationale	0.17	(-1.69, 0.09)	-1.69	0.0918
Collaboration	0.80	(-0.75, 0.46)	-0.75	0.4552
Teacher’s Role	1.11	(0.14, 0.89)	0.14	0.8885
Student’s Role	2.54	(1.59, 0.11)	1.59	0.1126
Outside Resources	2.14	(1.5, 0.13)	1.50	0.133
Syllabus Tone	1.59	(0.73, 0.46)	0.73	0.4641
Syllabus Focus	2.89	(1.45, 0.15)	1.45	0.1476
Grades	0.38	(-0.99, 0.32)	-0.99	0.3217
Feedback Mechanisms	1.06	(0.1, 0.92)	0.10	0.9231
Evaluation	1.46	(0.47, 0.64)	0.47	0.6399
Learning Outcomes	0.79	(-0.46, 0.65)	-0.46	0.6463
Revision/Redoing	1.68	(1.04, 0.3)	1.04	0.2968
Syllabus Length	1.00	(-0.01, 0.99)	-0.01	0.9914
AIC = 79.71				

*Note*. The coefficients represent the increase/decrease in the odds of being in the small opportunity gap group for each of the 13 rubric items and syllabus length as model 1 variables while holding the other variables constant. We used AIC to choose the best model (lowest AIC).

**Table 4** presents the results of the second stage of our analysis, the logistic regression model that predicts the log odds of a syllabus coming from the small opportunity gap group based on the three rubric factors and the syllabus length. This model shows *Power and Control* is a significant predictor of a syllabus coming from a course-instructor pair from the small opportunity gap group; increasing the average *Power and Control* to be more learner-centered by 1 point is associated with 19.62 times increase in the odds of being in the small opportunity gap group.

**Table 4:** Model 2. Logistic regression with syllabus factors and syllabus length as covariates.

	Exponentiated Coefficients	95% Confidence Interval for the Odds Ratio	Test Statistic	p-value	
(Intercept)	0.04	(0, 1.22)	-1.85	0.0645	.
*Community*	0.50	(0.16, 1.55)	-1.21	0.2266	
*Power and Control*	19.62	(2.36, 162.83)	2.76	0.0058	*
*Evaluation and Assessment*	0.98	(0.15, 6.58)	-0.02	0.9822	
Syllabus Length	0.85	(0.65, 1.11)	-1.23	0.2198	
AIC = 67.64					

*Note*. The coefficients represent the odds of being in the small opportunity gap group for each of the 3 rubric factors and syllabus length while holding the other variables constant. We used AIC to choose the best model (lowest AIC).

The final stage of our analysis was running the best subsets logistic regressions in two steps and choosing the models with the lowest AIC. The top 5 models for each step (regressing the log odds of being in the small opportunity gap group on the subset of covariates) can be found in the supplementary materials (**S4 Table in S1 File**) along with the full description of the best subsets regression procedure. After executing the first step of the best subsets procedure, the model with the lowest AIC has Learning Rationale, Students Role, Outside Resources, and Syllabus Focus included as covariates and is presented in supplemental **S5 Table in S1 File**. Out of all the rubric items, the most significant predictors of the odds of the syllabus being in the small opportunity gap group are Learning Rationale, Student’s Role, Outside Resources, and Syllabus Focus. If we consider syllabi from course-instructor pairs with the same rubric scores for Student’s Role, Outside Resources, and Syllabus Focus, increasing the Learning Rationale is associated with a decrease in the odds of having a small opportunity gap. While only marginally significant (at the *α* = 0.10 level), an increase in each of the rubric scores for Students’ Role, Outside Resources, and Syllabus Focus to be more learner-centered is associated with greater odds of having a syllabus being in the small opportunity gap group. Students’ Role, Outside Resources, and Syllabus Focus are all components of the *Power and Control* rubric factor which we previously saw from **Table 4** to be a significant predictor of the odds of being in the small opportunity gap group. We also note that a stepwise logistic regression yields the same model as the one found in supplemental materials **S5 Table in S1 File**.

The final model (the result of step 2 of the best subsets) which is the model with the lowest AIC is presented in **Table 5**. It has two covariates (1) Learning Rationale, and (2) *Power and Control*. Increasing Learning Rationale, while holding *Power and Control* constant is associated with a decrease in the odds of being in the small opportunity gap group. If Learning Rationale remains unchanged, but *Power and Control* is increased, there is a 75% increase in the odds of being in the small opportunity gap group.

**Table 5:** Model 4: The logistic regression with the lowest AIC when considering the syllabus items from Model 3 (Learning Rationale, Student’s Role, Outside Resources, and Syllabus Focus), three syllabus factors and syllabus length.

	Exponentiated Coefficients	95% Confidence Interval for the Odds Ratio	Test Statistic	p-value	
(Intercept)	0.17	(0.01, 3.45)	-1.15	0.2492	
Learning Rationale	0.19	(0.05, 0.80)	-2.28	0.0229	*
*Power and Control*	1.75	(1.20, 2.54)	2.94	0.0033	.
AIC = 59.75					

*Note*. The coefficients represent the odds of being in the small opportunity gap group. The final model includes one syllabus item (Learning Rationale) and one syllabus factor (Power and Control). We used AIC to choose the best model (lowest AIC).

## Discussion

While considerable work is being undertaken to examine how to create more learner-centered STEM classroom spaces to address their reputation for having “chilly climates”, course syllabi have yet to be a significant part of this discussion. Previous studies have examined the extent to which a syllabus can be learner-centered [22, 28] as well as the positive impacts of learner-centered pedagogy on minoritized students in reducing opportunity gaps [14]. The current study complements these efforts by confirming a relationship between the learner-centeredness of a course syllabus and the size of the opportunity gap (**Fig 1**).

In addition to examining the extent to which syllabus learner-centeredness is related to opportunity gaps, we investigated which rubric components have the strongest relationship with course opportunity gaps. The *Power and Control* factor (composed of Teacher’s Role, Students’ Role, Outside Resources, Syllabus Tone, Syllabus Focus rubric items) showed significant association with course-instructor pairs with small opportunity gaps (**Table 5**). Further investigation showed that Student’s Role, Outside Resources, and Syllabus Focus (three out of five items under *Power and Control*) are mainly responsible for this finding. Higher scores for *Power and Control* represent syllabi that exhibit higher student responsibility when it comes to learning and generating knowledge, more use of outside resources for learning outside the classroom with independent investigation, and less focus on policies and procedures as opposed to course objectives and learning outcomes.

These findings are in alignment with previous work that explores student and instructor perspectives and behaviors within a course. Recent research on the effectiveness of resources in engineering college classes shows that students find textbooks to be the least effective resource provided to them because of textbooks’ complexity and formality which makes them less accessible [42]; however, external resources such as educational websites or material generated by the instructor are considered more useful [42]. Similarly, our data showed that syllabi that provided resources other than the textbook were slightly associated with course-instructor pairs with small opportunity gaps. Providing additional resources may emphasize students’ responsibility for their learning and increase students’ role in acquiring knowledge. In a learner-centered class, students are actively involved in the process of learning instead of a one-way transfer of information from the instructor to student [18]. Our findings suggest that syllabi (as a proxy for their corresponding courses) that require students to play a more significant role in advancing their learning can increase the odds of having small opportunity gaps compared to syllabi (and by proxy their corresponding courses) that do not. These findings support the importance of highlighting an increased students’ role in STEM course syllabi.

Research also suggests that syllabi that focus on course rules instead of student learning can be perceived negatively by students, signaling “an adversarial relationship between instructors and students” [30 p. 19]. Syllabi that sound “authoritative and rule-infested” may indicate a lack of shared power in the classroom and lead students to have an unfavorable perception of the course and instructor [26 p. 37], negatively impacting student engagement [30] and success, especially for minoritized students [32]. In contrast, syllabi that focus more on learning goals and outcomes and empower students by including them in decision making, giving them more choice, and sharing power in the learning community promote a shift towards learner-centeredness [18, 30]. Similarly, in our current work, Syllabus Focus, which characterizes the degree to which syllabi focus on student learning, marginally correlates with the odds of courses having small opportunity gaps.

Another rubric item related to course outcome gap size was Learning Rationale, which describes the degree to which assignments, activities, policies and procedures are connected to the course learning outcomes. Surprisingly, a higher Learning Rationale correlated with larger opportunity gaps. This might be related to the instructor’s perception of students and the need for a detailed Learning Rationale. If the syllabus is written with the assumption that students do not bring any previous knowledge, skills, or individual experiences to class and have no driving motivators, the inclusion of an extensive Learning Rationale in the syllabus might be needed to compensate for these deficiencies. This may limit students’ agency and choice. According to Deci et al., “a rationale that is personally meaningful to the target person can aid him or her in understanding why self-regulation of the activity would have personal utility” [43 p. 124]. It seems logical that providing a rationale for learning without changing the dynamic of power and control in a classroom may prevent this “personally meaningful” connection to the course, negatively impacting the student experience. Therefore, instructors cannot rationalize why it is important to learn a particular topic in a vacuum but must provide opportunities for students to bring in their own experiences as well in order to have a more learner-centered environment.

### Limitations and future directions

All the collected syllabi were from a single institution that enrolls nearly 30,000 undergraduates, is classified as an R1-institution by the United States’ Carnegie Classification of Institutes of Higher Education and is a US designated minority-serving institution. Thus, it is possible that our findings are limited to course sections and syllabi found at similar institutions within the US. However, as it enrolls a significant number (nearly 50%) of first-generation and low-income students, this population may also represent enrollees in less-research intensive universities or two-year institutions as well [44], making our findings more generalizable across a broader range of higher education institutions.

Our claims regarding the learner-centeredness of a course are also limited to the course syllabus and did not leverage any other data from the instructor, course, or students for this characterization. This potentially includes classroom observation data, faculty survey or interview data, or student survey or interview data, in order to capture the degree to which the practices or environment was deemed to be learner-centered. We believe that our study establishes a foundation upon which future work can directly capture instructor practices in the classroom or student perspectives regarding their course experience to determine the degree to which these data align with the course syllabus.

Our work also looks broadly across a variety of STEM courses and does not attempt to identify important syllabi characteristics in the context of specific STEM disciplines. We did this intentionally, as this work is a starting point for future studies but must acknowledge that considerable research has highlighted that the climate can vary widely between STEM fields in terms of inclusion for women, minoritized students, or first-generation students [10, 45]. As a result, future work with more intentional disciplinary representation will be necessary to determine whether this is the case. From an institutional perspective, discipline or individual specific findings could also help to create more targeted feedback or training to particular departments or instructors. Similarly, while the basis of this work was on opportunity gaps between students from minoritized and non-minoritized populations, examining the impacts of syllabi for students of other demographics groups, such as different genders, income status, or first-generation status, will also complement this study.

### Recommendations for administrators

The finding that syllabus learner-centeredness correlates with opportunity gaps has important implications for institutions of higher education that are aiming to increase inclusivity in their STEM programs. As opposed to work which focuses on “fixing” minoritized students [46, 47], a syllabus is a hallmark of a specific course section, as is the course climate for which it serves as a proxy. To help instructors create more learner-centered syllabi, it is vital that the appropriate professional development mechanisms are available for support [48], in that, altering one’s syllabus could serve as a gateway to reflect on one’s course policies and instructional practices and potentially result in the creation of more learner-centered spaces. Institutions also must reward faculty for the time dedicated to participating in these activities. It is well-known that research-intensive institutions value research productivity over teaching-related activities, and that time spent on teaching is often discouraged [49, 50]. It has also been acknowledged that the evaluation of teaching, particularly the reliance on student evaluations of teaching, often fails to capture instructor effectiveness in promoting inclusive learning environments [51–53]. We propose that the steps taken in this work can be replicated for instructor merit and promotion processes, and if scaffolded with the appropriate professional development activities, can be a means by which an institution encourages and rewards its instructors for better supporting their students. The exercise of constructing more learner-centered syllabi may drive instructors to become more reflective in their teaching, leading them to reconsider the pedagogical practices and policies that they implement in their courses.

## Supporting information

S1 File (DOCX)

## References

[1] Haveman R, Smeeding T. The role of higher education in social mobility. The Future of children. 2006 Oct 1:125–50. doi: 10.1353/foc.2006.0015 17036549

[2] Ma J, Pender M, Welch M. Education Pays 2016: The Benefits of Higher Education for Individuals and Society. Trends in Higher Education Series. College Board. 2016.

[3] U.S. Department of Education, National Center for Education Statistics (NCES). Table 302.60: Percentage of 18- to 24-year-olds enrolled in college, by level of institution and sex and race/ethnicity of student: 1970 through 2021. In U.S. Department of Education, National Center for Education Statistics (Ed.), Digest of Education Statistics. 2023 Mar 8. Retrieved from https://nces.ed.gov/programs/digest/d20/index.asp.

[4] Foma E. Impact of workplace diversity. Review of Integrative Business and Economics Research. 2014;3(1):382.

[5] Olson S, Riordan DG. Engage to excel: producing one million additional college graduates with degrees in science, technology, engineering, and mathematics. Report to the president. Executive office of the president. 2012 Feb.

[6] Daempfle PA. An analysis of the high attrition rates among first year college science, math, and engineering majors. Journal of College Student Retention: Research, Theory & Practice. 2003 May;5(1):37–52.

[7] Doolen TL, Long M. Identification of retention levers using a survey of engineering freshman attitudes at Oregon State University. European Journal of Engineering Education. 2007 Dec 1;32(6):721–34.

[8] Flynn DT. STEM field persistence: The impact of engagement on postsecondary STEM persistence for underrepresented minority students. Journal of Educational Issues. 2016;2(1):185–214.

[9] Swail WS. Retaining Minority Students in Higher Education: A Framework for Success. ASHE-ERIC Higher Education Report. Jossey-Bass Higher and Adult Education Series. Jossey-Bass, 989 Market Street, San Francisco, CA 94103–1741; 2003.

[10] Whitcomb KM, Cwik S, Singh C. Not all disadvantages are equal: Racial/ethnic minority students have largest disadvantage among demographic groups in both STEM and non-STEM GPA. AERA Open. 2021 Nov; 7:23328584211059823.

[11] Armbruster P, Patel M, Johnson E, Weiss M. Active learning and student-centered pedagogy improve student attitudes and performance in introductory biology. CBE—Life Sciences Education. 2009 Sep;8(3):203–13. doi: 10.1187/cbe.09-03-0025 19723815 PMC2736024

[12] Freeman S, Eddy SL, McDonough M, Smith MK, Okoroafor N, Jordt H, et al. Active learning increases student performance in science, engineering, and mathematics. Proceedings of the national academy of sciences. 2014 Jun 10;111(23):8410–5. doi: 10.1073/pnas.1319030111 24821756 PMC4060654

[13] Rainey K, Dancy M, Mickelson R, Stearns E, Moller S. A descriptive study of race and gender differences in how instructional style and perceived professor care influence decisions to major in STEM. International Journal of STEM Education. 2019 Dec;6(1):1–3. 10.1186/s40594-018-0115-6 PMC6310405 30631700

[14] Theobald EJ, Hill MJ, Tran E, Agrawal S, Arroyo EN, Behling S, et al. Active learning narrows achievement gaps for underrepresented students in undergraduate science, technology, engineering, and math. Proceedings of the National Academy of Sciences. 2020 Mar 24;117(12):6476–83. doi: 10.1073/pnas.1916903117 32152114 PMC7104254

[15] Vygotsky LS, Cole M. Mind in society: Development of higher psychological processes. Harvard university press; 1978.

[16] Dano-Hinosolango MA, Vedua-Dinagsao A. The impact of learner-centered teaching on students’ learning skills and strategies. International Journal for Cross-Disciplinary Subjects in Education. 2014;5(4):1813–7.

[17] Weimer M. Learner-centered teaching: Five key changes to practice. John Wiley & Sons; 2013 Jan 28.

[18] Wright GB. Student-centered learning in higher education. International journal of teaching and learning in higher education. 2011;23(1):92–7.

[19] Bell BS, Kozlowski SW. Toward a theory of learner-centered training design: An integrative framework of active learning. In Learning, training, and development in organizations. Routledge. 2009 Aug 6: 263–300.

[20] Smart KL, Csapo N. Learning by doing: Engaging students through learner-centered activities. Business Communication Quarterly. 2007 Dec;70(4):451–7.

[21] Ballen CJ, Wieman C, Salehi S, Searle JB, Zamudio KR. Enhancing diversity in undergraduate science: Self-efficacy drives performance gains with active learning. CBE—Life Sciences Education. 2017 Dec;16(4):ar56. doi: 10.1187/cbe.16-12-0344 29054921 PMC5749958

[22] Cullen R, Harris M. Assessing learner‐centredness through course syllabi. Assessment & Evaluation in Higher Education. 2009 Feb 1;34(1):115–25.

[23] Goodwin A, Chittle L, Dixon JC, Andrews DM. Taking stock and effecting change: curriculum evaluation through a review of course syllabi. Assessment & Evaluation in Higher Education. 2018 Aug 18;43(6):855–66.

[24] Palmer MS, Bach DJ, Streifer AC. Measuring the promise: A learning‐focused syllabus rubric. To Improve the Academy. 2014 Sep;33(1):14–36.

[25] Stanny C, Gonzalez M, McGowan B. Assessing the culture of teaching and learning through a syllabus review. Assessment & Evaluation in Higher Education. 2015 Oct 3;40(7):898–913.

[26] Palmer MS, Wheeler LB, Aneece I. Does the document matter? The evolving role of syllabi in higher education. Change: The Magazine of Higher Learning. 2016 Jul 3;48(4):36–47.

[27] Eberly MB, Newton SE, Wiggins RA. The syllabus as a tool for student-centered learning. The Journal of General Education. 2001 Jan 1:56–74.

[28] Richmond AS, Morgan RK, Slattery JM, Mitchell NG, Cooper AG. Project syllabus: An exploratory study of learner-centered syllabi. Teaching of Psychology. 2019 Jan;46(1):6–15.

[29] Kim Y, Ekachai DG. Exploring the effects of different online syllabus formats on student engagement and course-taking intentions. College Teaching. 2020 Oct 1;68(4):176–86.

[30] DiClementi JD, Handelsman MM. Empowering students: Class-generated course rules. Teaching of Psychology. 2005 Feb 1;32(1):18–21.

[31] Harnish RJ, Bridges KR. Effect of syllabus tone: Students’ perceptions of instructor and course. Social Psychology of Education. 2011 Sep; 14:319–30.

[32] Kuh GD, Kinzie J, Cruce T, Shoup R, Gonyea RM. Connecting the dots: Multi-faceted analyses of the relationships between student engagement results from the NSSE, and the institutional practices and conditions that foster student success. Indiana University Center for Postsecondary Research; 2007.

[33] Denaro K, Dennin K, Dennin M, Sato B. Identifying systemic inequity in higher education and opportunities for improvement. PloS one. 2022 Apr 8;17(4): e0264059. doi: 10.1371/journal.pone.0264059 35395005 PMC8993022

[34] Dyer JM, Suh EK, Posey B, Owens S. Laying bare the foundations: Examining and confronting language expectations in a college syllabus. In Beyond Equity at Community Colleges. Routledge. 2022 Jun 15: 30–47.

[35] Khoja SA, Sana F, Karim A, Rehman AA. Implementing constructivist pedagogical model in dynamic distance learning framework. In Wireless Networks, Information Processing and Systems: International Multi Topic Conference, IMTIC 2008 Jamshoro, Pakistan, April 11–12, 2008 Revised Selected Papers 2009 (pp. 191–201). Springer Berlin Heidelberg.

[36] Gamer M, Lemon J, Singh IF. irr: various coefficients of interrater reliability and agreement. 2019. R package version 0.84. 2017;1(6).

[37] Rizopoulos D. ltm: An R package for latent variable modeling and item response analysis. Journal of statistical software. 2007; 17:1–25.

[38] Landis JR, Koch GG. The measurement of observer agreement for categorical data. biometrics. 1977 Mar 1:159–74. 843571

[39] Team RC. R: A language and environment for statistical computing. R Foundation for Statistical Computing. 2021.

[40] Venables WN, Ripley BD. Modern Applied Statistics with S, Springer, New York: ISBN 0-387-95457-0. 2002

[41] McLeod AI, Xu C, Lai Y. bestglm: Best subset GLM and regression utilities. R package version 0.37. 2020;3.

[42] Maclaren P. How is that done? Student views on resources used outside the engineering classroom. European Journal of Engineering Education. 2018 Jul 4;43(4):620–37.

[43] Deci EL, Eghrari H, Patrick BC, Leone DR. Facilitating internalization: The self‐determination theory perspective. Journal of personality. 1994 Mar;62(1):119–42. doi: 10.1111/j.1467-6494.1994.tb00797.x 8169757

[44] Zusman A. Challenges facing higher education in the twenty-first century. American higher education in the twenty-first century: Social, political, and economic challenges. 2005; 2:115–60.

[45] Eddy SL, Brownell SE. Beneath the numbers: A review of gender disparities in undergraduate education across science, technology, engineering, and math disciplines. Physical Review Physics Education Research. 2016 Aug 1;12(2):020106.

[46] Harper SR. An anti‐deficit achievement framework for research on students of color in STEM. New directions for institutional research. 2010 Dec;2010(148):63–74.

[47] McGee EO. Devalued Black and Latino racial identities: A by-product of STEM college culture?. American Educational Research Journal. 2016 Dec;53(6):1626–62.

[48] Goos M, Dole S, Makar K. Designing professional development to support teachers’ learning in complex environments. Mathematics Teacher Education and Development. 2007; 8:23–47.

[49] Hardré P, Cox M. Evaluating faculty work: Expectations and standards of faculty performance in research universities. Research Papers in Education. 2009 Dec 1;24(4):383–419.

[50] Parker J. Comparing research and teaching in university promotion criteria. Higher education quarterly. 2008 Jul;62(3):237–51.

[51] Boring A, Ottoboni K. Student evaluations of teaching (mostly) do not measure teaching effectiveness. ScienceOpen research. 2016 Jan 7.

[52] Dewsbury B, Brame CJ. Inclusive teaching. CBE—Life Sciences Education. 2019;18(2): fe2. doi: 10.1187/cbe.19-01-0021 31025917 PMC7058128

[53] Radchenko N. Student evaluations of teaching: Unidimensionality, subjectivity, and biases. Education Economics. 2020 Nov 1;28(6):549–66.

