# Supplementary material for: How syllabi relate to outcomes in higher education: A study of syllabi learner-centeredness and grade inequities in STEM
Source: PLoS One. 2024 Apr 17;19(4):e0301331. doi: 10.1371/journal.pone.0301331 (PMC11023273; doi:10.1371/journal.pone.0301331)
Supplement: S1 File — (DOCX) [file pone.0301331.s001.docx]

**Supplemental Figure S1**

*Histogram of the size of the opportunity gap for* $n = 50$ *course-instructor pairs*


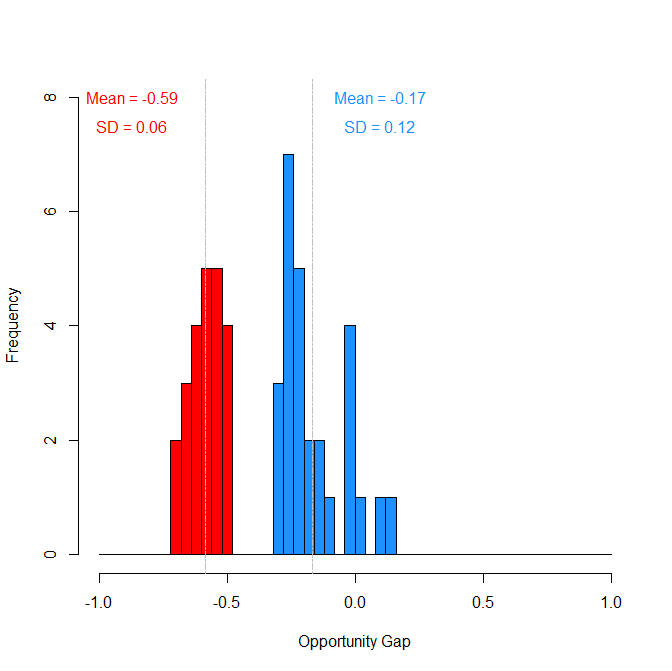


*Note.* Red indicates syllabi in the large opportunity gap group. Blue indicates syllabi in the small opportunity gap group.

**Supplemental Table S1**

*Rubric for syllabus evaluation*


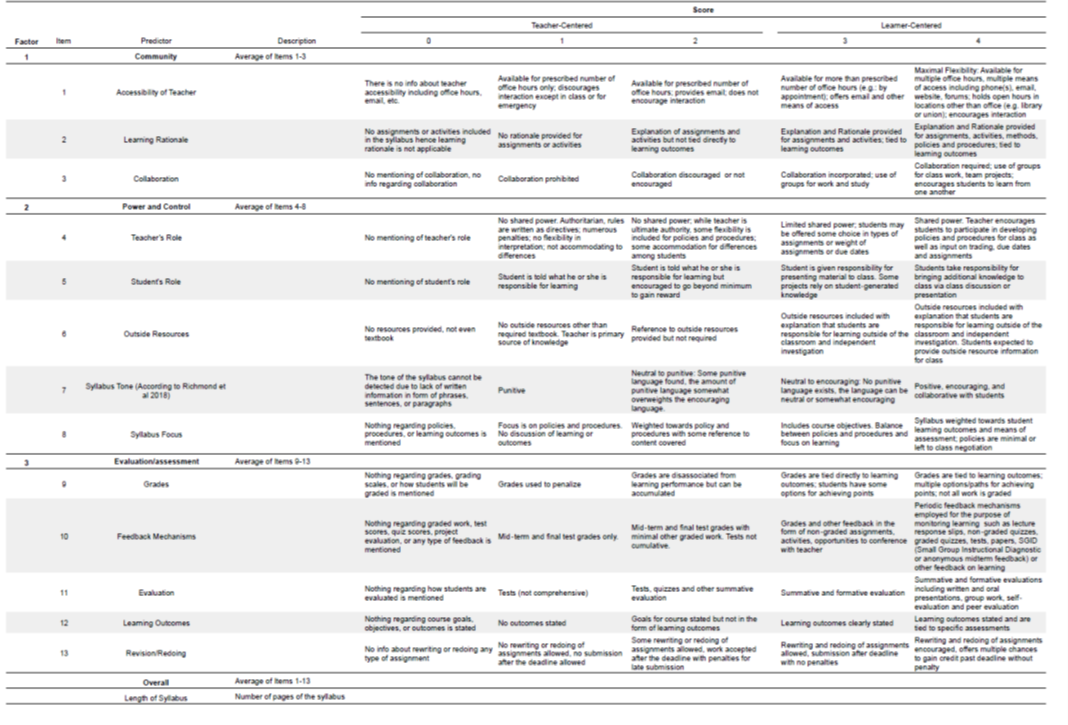


*Note.* The description for the 3 factors and 13 items is provided along with the explanation for scoring each rubric item.

**Supplemental Table S2**

*Correlation matrix for the rubric items*

|  |  |  |  |  |  |  |  |  |  |  |  |  |  |  |  |  |
| --- | --- | --- | --- | --- | --- | --- | --- | --- | --- | --- | --- | --- | --- | --- | --- | --- |
|  | *Community* | | |  | *Power and Control* | | | | |  | *Evaluation and Assessment* | | | | | |
|  | Accessibility of Teacher | Learning Rationale | Collaboration |  | Teacher’s Role | Student’s Role | Outside Resources | Syllabus Tone | Syllabus Focus |  | Grades | Feedback Mechanisms | Evaluation | Learning Outcomes | Revision/Redoing |  |
| *Community* |  | **--** |  |  |  |  | **0.51**** |  |  |  |  |  | **0.54**** |  |  |  |
| Accessibility of Teacher | -- | 0.22 | 0.00 |  | 0.24 | 0.04 | 0.38** | 0.20 | -0.09 |  | 0.07 | 0.25 | -0.01 | -0.01 | 0.10 |  |
| Learning Rationale |  | -- | 0.30* |  | 0.40** | 0.18 | 0.25 | 0.13 | 0.24 |  | 0.38** | 0.51** | 0.41** | -0.11 | 0.37** |  |
| Collaboration |  |  | -- |  | 0.13 | 0.41** | 0.27 | 0.10 | 0.13 |  | 0.32* | 0.44** | 0.70** | 0.05 | 0.26 |  |
| *Power and Control* |  |  |  |  |  |  | **--** |  |  |  |  |  | **0.55**** |  |  |  |
| Teacher’s Role |  |  |  |  | -- | 0.00 | 0.10 | 0.18 | 0.19 |  | 0.31* | 0.11 | 0.16 | -0.09 | 0.26 |  |
| Student’s Role |  |  |  |  |  | -- | 0.21 | 0.03 | 0.35* |  | 0.26 | 0.20 | 0.50** | 0.09 | 0.20 |  |
| Outside Resources |  |  |  |  |  |  | -- | 0.03 | -0.10 |  | 0.14 | 0.42** | 0.21 | -0.08 | 0.35* |  |
| Syllabus Tone |  |  |  |  |  |  |  | -- | 0.42** |  | 0.27 | 0.19 | 0.12 | 0.27 | -0.10 |  |
| Syllabus Focus |  |  |  |  |  |  |  |  | -- |  | 0.35* | 0.16 | 0.21 | 0.57** | -0.01 |  |
| *Evaluation/ Assessment* |  |  |  |  |  |  |  |  |  |  |  |  | **--** |  |  |  |
| Grades |  |  |  |  |  |  |  |  |  |  | -- | 0.39** | 0.48** | 0.24 | 0.18 |  |
| Feedback Mechanisms |  |  |  |  |  |  |  |  |  |  |  | -- | 0.50** | 0.06 | 0.12 |  |
| Evaluation |  |  |  |  |  |  |  |  |  |  |  |  | -- | 0.04 | 0.32* |  |
| Learning Outcomes |  |  |  |  |  |  |  |  |  |  |  |  |  | -- | -0.17 |  |
| Revision/Redoing |  |  |  |  |  |  |  |  |  |  |  |  |  |  | -- |  |
| *p<0.05.**p< 0.01 |  |  |  |  |  |  |  |  |  |  |  |  |  |  |  |  |

**Supplemental Table S3**

*Rubric* *summary statistics*

|  |  |  | Mean (SD) | |  | Median (IQR) | |
| --- | --- | --- | --- | --- | --- | --- | --- |
| **Factor** | Item | Predictor | Large opportunity gap group | Small opportunity gap group |  | Large opportunity gap group | Small opportunity gap group |
| **1** |  | *Community* | 1.90 (0.69) | 1.91 (0.84) |  | 2.00 (0.83) | 2.00 (1.33) |
|  | 1 | Accessibility of Teacher | 1.83 (1.34) | 1.96 (1.06) |  | 2.00 (3.00) | 2.00 (1.50) |
|  | 2 | Learning Rationale | 2.13 (0.69) | 1.96 (0.52) |  | 2.00 (1.00) | 2.00 (0.00) |
|  | 3 | Collaboration | 1.74 (1.48) | 1.81 (1.75) |  | 3.00 (3.00) | 3.00 (3.00) |
| **2** |  | *Power and Control* | 1.72 (0.43) | 2.07 (0.48) |  | 1.60 (0.70) | 2.00 (0.50) |
|  | 4 | Teacher’s Role | 1.57 (0.66) | 1.52 (0.70) |  | 2.00 (1.00) | 2.00 (1.00) |
|  | 5 | Student’s Role | 1.22 (0.52) | 1.81 (0.96) |  | 1.00 (0.50) | 2.00 (2.00) |
|  | 6 | Outside Sources | 1.70 (0.82) | 2.15 (1.13) |  | 1.00 (1.00) | 2.00 (2.00) |
|  | 7 | Syllabus Tone | 2.30 (0.76) | 2.56 (0.80) |  | 2.00 (1.00) | 3.00 (1.00) |
|  | 8 | Syllabus Focus | 1.83 (0.83) | 2.30 (0.95) |  | 2.00 (1.00) | 2.00 (1.00) |
| **3** |  | *Evaluation/Assessment* | 1.97 (0.34) | 2.10 (0.56) |  | 1.80 (0.50) | 2.20 (0.50) |
|  | 9 | Grades | 2.00 (0.43) | 2.04 (0.71) |  | 2.00 (0.00) | 2.00 (0.00) |
|  | 10 | Feedback Mechanisms | 2.30 (0.47) | 2.30 (1.07) |  | 2.00 (1.00) | 3.00 (1.00) |
|  | 11 | Evaluation | 2.70 (0.47) | 2.89 (0.93) |  | 3.00 (1.00) | 3.00 (1.00) |
|  | 12 | Learning Outcomes | 2.04 (0.98) | 2.22 (0.89) |  | 2.00 (2.00) | 2.00 (1.50) |
|  | 13 | Revision/Redoing | 0.78 (0.80) | 1.07 (0.92) |  | 1.00 (1.00) | 1.00 (2.00) |
|  |  | **Overall** | 1.86 (0.38) | 2.05 (0.52) |  | 1.85 (0.54) | 2.08 (0.62) |
|  |  | Length of Syllabus | 4.91 (3.67) | 4.85 (2.86) |  | 4.00 (2.00) | 4.00 (3.00) |
|  |  | n | 23 | 27 |  | 23 | 27 |

*Note.* The mean, median, and interquartile range (IQR) for each one of the rubric items, factors, and length of syllabus is provided for both small and large opportunity gap groups.

**Supplemental Table S4**

*The top 5 models regressing the log odds of being in the small opportunity gap group.*

| Step 1: |  |  |  |  |  |  |
| --- | --- | --- | --- | --- | --- | --- |
|  |  | Model | | | | |
| Covariate |  | A | B | C | D | E |
| Accessibility of Teacher |  | -- | -- | -- | -- | -- |
| Learning Rationale |  | TRUE | TRUE | TRUE | TRUE | TRUE |
| Collaboration |  | -- | -- | -- | -- | -- |
| Teacher’s Role |  | -- | -- | -- | -- | -- |
| Student’s Role |  | TRUE | TRUE | TRUE | TRUE | TRUE |
| Outside Resources |  | TRUE | TRUE | TRUE | TRUE | TRUE |
| Syllabus Tone |  | -- | -- | -- | -- | -- |
| Syllabus Focus |  | TRUE | TRUE | TRUE | TRUE | TRUE |
| Grades |  | -- | -- | TRUE | -- | -- |
| Feedback Mechanisms |  | -- | -- | -- | -- | -- |
| Evaluation |  | -- | -- | -- | -- | -- |
| Learning Outcomes |  | -- | -- | -- | -- | TRUE |
| Revision/Redoing |  | -- | TRUE | -- | -- | -- |
| Syllabus Length |  | -- | -- | -- | TRUE | -- |
| AIC |  | 61.64 | 62.41 | 62.75 | 62.90 | 62.94 |
|  |  |  |  |  |  |  |
| Step 2: |  |  |  |  |  |  |
|  |  | Model | | | | |
| Covariate |  | A | B | C | D | E |
| Learning Rationale |  | TRUE | TRUE | TRUE | TRUE | TRUE |
| Student’s Role |  | -- | TRUE | -- | -- | -- |
| Outside Resources |  | -- | -- | -- | -- | TRUE |
| Syllabus Focus |  | -- | -- | -- | -- | -- |
| *Community* |  | -- | -- | TRUE | -- | -- |
| *Power and Control* |  | TRUE | TRUE | TRUE | TRUE | TRUE |
| *Evaluation and Assessment* |  | -- | -- | -- | -- | -- |
| Syllabus Length |  | -- | -- | -- | TRUE | -- |
| AIC |  | 59.75 | 60.46 | 61.10 | 61.21 | 61.58 |

*Note.* Step 1: The first step of the best subset logistic regression ran every combination of models using syllabus items and syllabus length as the predictors. That is, this step considered models with 1-14 predictors (a total of 16,383 possible models). The model with the lowest AIC (model 3) has Learning Rationale, Students Role, Outside Resources, and Syllabus Focus included as covariates and is presented in supplemental materials Table A5.

Step 2: In the step above, we ran every combination of predictors from the previous step’s model (Learning Rationale, Student’s Role, Outside Resources, and Syllabus Focus), syllabus factors, and syllabus length for models with 1-8 predictors (a total of 255 possible models). The final model (the model with the lowest AIC) has two covariates (1) Learning Rationale, and (2) Power and Control and is presented in Table 4.

**Supplemental Table S5**

*Model 3. The logistic regression model with the lowest AIC when considering syllabus items and syllabus length was the one that included 4 rubric items (Learning Rationale, Student’s Role, Outside Resources, and syllabus focus)*

|  |  |  |  |  |  |
| --- | --- | --- | --- | --- | --- |
|  | Exponentiated Coefficients | 95% Confidence Interval for the Odds Ratio | Test Statistic | p-value |  |
| (Intercept) | 0.30 | (0.02, 4.78) | -0.85 | 0.3935 |  |
| Learning Rationale | 0.23 | (0.06, 0.90) | -2.11 | 0.0345 | * |
| Student’s Role | 2.35 | (0.93, 5.99) | 1.80 | 0.0722 | . |
| Outside Resources | 2.12 | (0.97, 4.63) | 1.89 | 0.0582 | . |
| Syllabus Focus | 2.25 | (0.91, 5.58) | 1.75 | 0.0793 | . |
| AIC = 61.64 |  |  |  |  |  |

*Note*. The coefficients represent the increase/decrease in the odds of being in the small opportunity gap group.
